# Effect of Maraviroc Intensification on HIV-1-Specific T Cell Immunity in Recently HIV-1-Infected Individuals

**DOI:** 10.1371/journal.pone.0087334

**Published:** 2014-01-27

**Authors:** Ai Kawana-Tachikawa, Josep M. Llibre, Isabel Bravo, Roser Escrig, Beatriz Mothe, Jordi Puig, Maria C. Puertas, Javier Martinez-Picado, Julia Blanco, Christian Manzardo, Jose M. Miro, Aikichi Iwamoto, Anton L. Pozniak, Jose M. Gatell, Bonaventura Clotet, Christian Brander, the MARAVIBOOST investigators

**Affiliations:** 1 Irsicaixa AIDS Research Institute – HIVACAT, Autonomous University of Barcelona, Badalona, Spain; 2 Division of Infectious Diseases, Advanced Clinical Research Center, The Institute of Medical Science, The University of Tokyo, Tokyo, Japan; 3 Lluita contra la SIDA Foundation, HIV Unit, University Hospital Germans Trias i Pujol, Badalona, UAB, Badalona, Spain; 4 University of Vic, Vic, Spain; 5 Institució Catalana de Recerca i Estudis Avancats (ICREA), Barcelona, Spain; 6 Hospital Clínic-IDIBAPS, University of Barcelona, Barcelona, Spain; 7 HIV/GUM Department, Chelsea and Westminster Hospital, London, United Kingdom; New York University, United States of America

## Abstract

**Background:**

The effect of maraviroc on the maintenance and the function of HIV-1-specific T cell responses remains unknown.

**Methods:**

Subjects recently infected with HIV-1 were randomized to receive anti-retroviral treatment with or without maraviroc intensification for 48 weeks, and were monitored up to week 60. PBMC and *in vitro*-expanded T cells were tested for responses to the entire HIV proteome by ELISpot analyses. Intracellular cytokine staining assays were conducted to monitor the (poly)-functionality of HIV-1-specific T cells. Analyses were performed at baseline and week 24 after treatment start, and at week 60 (3 months after maraviroc discontinuation).

**Results:**

Maraviroc intensification was associated with a slower decay of virus-specific T cell responses over time compared to the non-intensified regimen in both direct *ex-vivo* as well as *in in-vitro* expanded cells. The effector function profiles of virus-specific CD8^+^ T cells were indistinguishable between the two arms and did not change over time between the groups.

**Conclusions:**

Maraviroc did not negatively impact any of the measured parameters, but was rather associated with a prolonged maintenance of HIV-1-specific T cell responses. Maraviroc, in addition to its original effect as viral entry inhibitor, may provide an additional benefit on the maintenance of virus-specific T cells which may be especially important for future viral eradication strategies.

## Introduction

Maraviroc is an antiretroviral agent that blocks HIV-1 entry by binding the virus' coreceptor CCR5. Given its molecular target, maraviroc treatment may modulate the natural expression and function of CCR5, and negatively affect chemotaxis and effector function of Th1-type CD4^+^ T cell and memory CD8^+^ T cells. Maraviroc may have additional immunomodulatory effects by blocking the binding of the natural ligands of CCR5 (MIP-1α, MIP-1β and, RANTES), yet little data exist on how maraviroc may interfere with the cellular host immunity, especially the one directed against HIV-1.

While CCR5 deficiency (in the form of a 32 base-pair homozygous deletion) can mediate resistance to HIV-1 infection [1]–[3], it also has the potential to impair control of other viral infections, such as West Nile virus (WNV), both in mouse and humans [4], [5]. In particular, murine T cells lacking CCR5 expression have been shown to secrete lower amounts of IL-2 compared to CCR5^+^ T cells, and a similar phenotype has been observed in T cells from humans expressing the CCR5-∶32 mutation [6]. Furthermore, CD8^+^ T cell exhaustion during chronic Lymphocytic choriomeningitis virus (LCMV) infection is more severe in the absence of RANTES, one of the natural CCR5 ligands [7]. Thus, although CCR5-∶32 homozygosity does not seem to negatively affect humans, blocking its function by agents like maraviroc may negatively affect immune responses, including T cell responses to HIV-1.

In previous clinical trials, treatment with maraviroc has been shown to result in more extensive increases in CD4 counts in treatment-naïve and -experienced subjects, though the mechanisms involved remain unknown [8]–[11]. In addition, some studies have indicated that adding maraviroc to suppressive combination antiretroviral treatment (cART) reduces markers of immune activation [12]–[15]. Also, *in vitro* exposure to maraviroc decreases some markers of immune activation on T lymphocytes [16]. While these findings suggest that maraviroc may have beneficial effects on global host immune status, maraviroc has also been found to increase T cell activation both in gut and peripheral blood [17]. Thus, it is still controversial whether maraviroc has net immunological benefits or disadvantages on host cellular immune responses. In addition, the impact of maraviroc on antigen-specific T cell responses, especially towards HIV-1-derived antigens, has not been assessed, despite its potential implications with regards to immune interventions, particularly therapeutic vaccination in maraviroc treated subjects. To address these issues, we analyzed in a longitudinal study the effects of cART versus maraviroc–intensified cART on the maintenance (breadth, magnitude and specificity) of HIV-1-specific T cell responses, their differentiation potential and their polyfunctionality.

## Materials and Methods

### Study design

The present study was performed as sub-study of the Maraviboost study (ClinicalTrials.gov Identifier: NCT00808002). The Maraviboost study was a multi-center, randomized, open-label, phase III clinical trial. The main goal of the parental clinical trial was to assess whether intensification with maraviroc in recently HIV-1 infected patients with standard triple therapy could accelerate the decay of the HIV-1 reservoir [18]. Thirty subjects recently infected with CCR5-tropic HIV-1 (subtype B) were recruited and randomized into 2 groups (n = 15 each), one being treated with triple therapy consisting of Raltegravir (RAL) plus Tenofovir (TDF)/Emtricitabine (FTC) alone while the second group received additionally maraviroc (MVC) intensification for the first 48 weeks in the trial. The primary end point of the main study was week 48, but patients were followed until week 72 if possible. Frozen PBMC from pre-defined time points before starting cART (baseline, BL), 24 weeks after study initiation, and 12 weeks after maraviroc discontinuation (week 60), were analyzed in the present study. One patient without maraviroc intensification, who dropped out the study because of adherence problem, was excluded from the analysis. Three patients (01028, 01039, 23012) were lost at week 24 (n = 1) or 36 (n = 2), respectively. All patients received RAL plus TDF/FTC after week 48 except 4 patients (01021, 01031, 01034, 01043), who changed their anti-HIV drug regimen. Of the 29 individuals, peripheral blood mononuclear cells (PBMC) from at least one time point were available for 13 patients with maraviroc intensification (MVC arm) and 14 patients without MVC intensification (Control, CNT arm, Table 1). The study was approved by the ethics committee of Hospital Germans Trias i Pujol, Badalona, Spain. All patients gave their written informed consent before enrolling in the study.

**Table 1:** Characteristics of participants.

patient ID	age		Estimated duration from infection	baseline			week24			week60		
				VL	CD4	CD8	VL	CD4	CD8	VL	CD4	CD8
	(year-old)		(months)	(copies/ml)	(cells/*μ*l)	(cells/*μ*l)	(copies/ml)	(cells/*μ*l)	(cells/*μ*l)	(copies/ml)	(cells/*μ*l)	(cells/*μ*l)
Control group (n = 14)												
01022	28	6.2		61,000	606	873	50	843	731	50	741	606
01025	32	5.8		^a^36,000	287	691	^b^50	446	809	50	432	527
01028	26	3.1		490,000	366	1,893	56	518	900	^c^not determined		
01030	21	4.0		19,000	297	1,124	50	435	464	50	650	650
01032	32	4.5		^a^200,000	372	1,830	200	478	956	50	580	825
01036	42	5.1		40,000	273	1,034	50	352	641	50	429	617
01037	40	3.2		^a^26,000	589	1,326	50	533	999	50	556	641
01039	26	8.6		^a^63,000	450	1,125	50	654	1,162	^c^not determined		
01040	50	2.8		1,500,000	379	2,245	^b^230	601	631	50	620	589
01044	35	3.8		^a^540,000	454	1,127	50	962	1,683	50	396	834
23010	26	4.7		^b^1,091	629	1,204	^b^50	624	559	50	649	593
23012	38	4.0		10,738	656	579	50	688	615	^c^not determined		
23013	39	4.1		30,210	492	1,624	94	644	984	50	624	635
23019	32	6.5		8,497	620	1,033	^b^50	688	899	50	543	688
median	32	4.3		38,000	452	1,126	50	613	854	50	580	635
(itnterquartile range)	(26–39)	(3.7–5.9)		(16,935–272,5000)	(349–610)	(993–1,676)	(50–65)	(470–688)	(627–989)	(50–50)	(432–649)	(593–688)
MVC intensified group (n = 13)												
01021	39	5.1		46,000	649	1,750	50	954	1,371	^c^not determined		
01027	32	6.6		120,000	558	888	50	767	684	50	941	811
01034	33	4.6		12,000	310	496	55	285	498	^c^not determined		
01035	35	4.2		320,000	384	1,024	50	706	1,169	50	984	1,312
01041	34	2.0		160,000	619	1,695	50	1,034	853	50	542	383
01042	33	2.3		^a^140,000	280	1,595	50	602	1,228	50	654	782
01043	37	2.3		320,000	617	1,163	50	679	928	^c^not determined		
01045	49	1.1		^a^470,000	639	408	60	1,077	661	50	832	407
23005	28	5.4		5,666	421	666	^b^50	572	717	^b^50	500	521
23007	26	2.2		149,556	641	1,124	50	770	1,183	50	839	896
23011	31	5.8		11,081	454	1,473	^b^50	680	1,214	50	843	1,448
23015	42	8.2		54,216	283	2,384	61	492	1,638	50	743	1,974
23016	35	6.8		51,478	397	719	50	653	642	50	515	480
median	34	4.6		120,000	454	1,124	50	680	928	50	788	797
(itnterquartile range)	(31–38)	(2.3–6.2)		(29,000–240,000)	(347–629)	(693–1,645)	(50–53)	(587–863)	(673–1,221)	(50–50)	(535–868)	(462–1,346)
***P value***				***0.5541***	***0.6623***	***0.5603***	***0.5925***	***0.1263***	***0.2541***		***0.0378***	***0.6472***

a: data from the closest previous timpoint for VL, CD4, CD8. The gap was 14–35 days.

b: not analyzed in this study because of sample limitation.

c: not determined because of lost patients.

### Flow cytometry for T cell phenotype analysis

PBMC were thawed and rested overnight at 37°C in RPMI1640 supplemented with 10% heat-inactivated FCS, 100 U/ml penicillin, 100*μ*g/ml streptomycin, and 2 mM glutamine (R10). The following day, the cells were stained with LIVE/DEAD Fixable Dead Cell Stain Kits (Invitrogen), washed and stained with the following antibodies: anti-CD3-APC-Cy7, anti-CD4-V450, anti-CD8-PE-Cy7, anti-CD45RA-APC (BD Biosience), and anti-CCR7-PE (e-BioScience). The cells were washed and fixed with 1% Formaldehyde in PBS. All data were collected on a BD LSR II flow cytometer (BD Biosience) and analyzed using FlowJo 8.7.7 (TreeStar).

### Peptides

A set of 410 overlapping-peptides (OLPs) was used to screen for HIV-specific T-cell responses [19]. The peptides spanned all HIV-1 proteins and were based on the HIV clade B consensus sequence of 2001, available at the Los Alamos National Laboratory HIV immunology database. For ELISpot analyses, peptides were used in a matrix layout of 6–12 peptides per pool for comprehensive screening as previously described [19]. Reconfirmations of all positive wells in the matrix screen were performed the following day on a single-peptide base. For multi-functional analysis by flow cytometry, peptide pools were used that contained peptides spanning either full-length Gag, Protease, RT, IN, gp120, gp41, or Nef. Peptides spanning Tat, Rev, Vif, Vpr, and Vpu were combined into one peptide pool (accessory proteins peptide pool. “Acc”).

### IFN-γ ELISpot assay using ex-vivo PBMC and in-vitro expanded T cells

Thawed PBMC were rested for 3 hrs at 37°C in R10. If sufficient PBMC were recovered, thawed cells were used directly in IFN-γ ELISpot assays (11 and 7 samples at baseline, 6 and 7 samples at week 24, and 8 and 7 samples at week 60 in the CNT and MVC arm, respectively). In addition, 1×10^6^ thawed cells were stimulated with an anti-CD3 monoclonal antibody and cultured for 2–4 weeks in R10 supplemented with 50 U/ml of recombinant IL-2 [20]. Before use in ELISpot assays, the expanded cells were washed twice with R10 and incubated overnight at 37°C in the absence of IL-2. Per well, 75,000–100,000 cells were used and peptides were added as in the direct ex-vivo assay. Thresholds for positive responses were defined as 1) at least five spots (50–66 SFC/10^6^ PBMC) per well, 2) as responses exceeding the mean of negative wells plus 3 standard deviation and 3) responses exceeding three times the mean of negative (no peptide) wells; whichever was the highest. For reconfirmation ELISpot, the remaining cells and cells from negative wells from initial matrix screens were recycled as previously described [20].

### Flow cytometric analysis of CD8^+^ T cell function

Thawed PBMC were rested overnight at 37°C in R10. The following day, costimulatory antibodies (anti-CD28 and anti-CD49d at 1*μ*g/ml; BD Biosciences) and monensin (GolgiStop; BD Bioscience) were added, and cells were stimulated with the different peptide pools (5*μ*g/ml per peptide) as indicated. A negative (no peptide) and a positive control (phorbol-12-myristate-13-acetate (PMA at 10 ng/ml and ionomycin, 1*μ*M) were included in each assay. Following incubation for 6 hrs, the cells were washed with PBS containing 1% FCS and the fluorescent reactive dye (Invitrogen) for dead cells was added. Cells were washed again, and stained with anti-CD3-V450, anti-CD8-PerCP, and anti-CD107a-PE (BD BioScience). Following washing, the cells were fixed and permeabilized using Fix & Perm cell permeabilization reagents (Invitrogen). The cells were then stained with anti-MIP-1β-FITC, anti-IL-2-PE-Cy7, anti-IFN-γ-APC (BD Bioscience). Data were collected on a BD LSR II flow cytometer (BD Biosience) and analyzed using FlowJo 8.7.7 (TreeStar). After gating for each effector function, a Boolean gate platform was used to create the full array of possible combinations and SPICE software (version 5.22) was used to analyze the polychromatic flow cytometry data. We applied a threshold for positive responses using negative values distribution after background subtraction (i.e. unstimulated cultures), as previously described [21].

### Statistical analyses

Statistical analyses were performed using Graph Pad Prism 5.0. The results are given as medians and interquartile range (IQR) as indicated. Mann-Whitney test and Wilcoxon matched paired test were used for unpaired and paired comparisons, respectively. For multiple comparison analysis, we performed Bonferroni correction. Correlations between *ex-vivo* and *in-vitro* ELISpot data were analyzed by using Spearman's rank correlation coefficient, and linear regression analysis.

## Results

### Changes in CD4^+^ and CD8^+^ T cell count and their differentiation status

**Figure 1:**
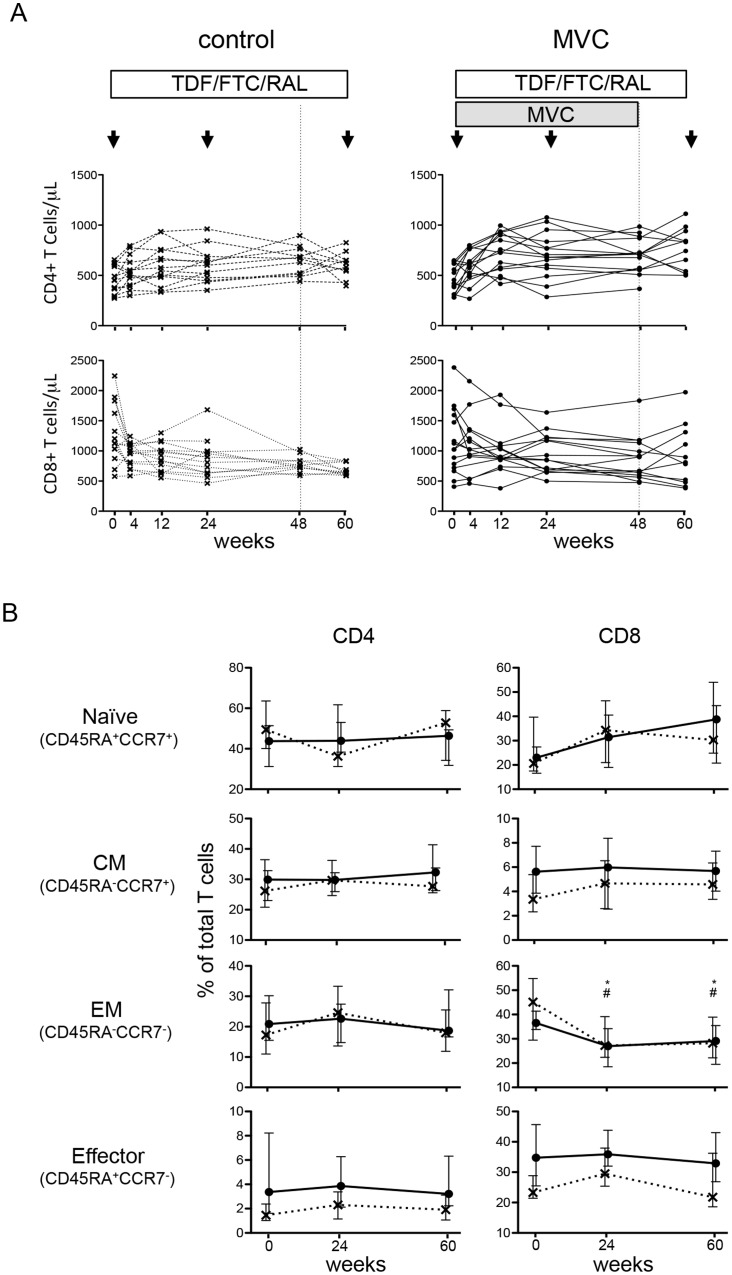
Differentiation status in CD4^+^ and CD8^+^ T cells. A. Changes of CD4^+^ and CD8^+^ T cell count in each subject. B. The proportion of naïve (CD45RA^+^/CCR7^+^), central memory (CM, CD45RA^−^/CCR7^+^), effector memory (EM, CD45RA^−^/CCR7^−^), and Terminal effector memory (T_EMRA_, CD45RA^+^/CCR7^−^) cells among CD4^+^ and CD8^+^ T cells in the control (cross and hatched line) and MVC arm (circle and solid line). The median and interquartile range (vertical line) are shown. Stars (control) and hatches (MVC arm) above the lines indicate significant differences relative to baseline values (p<0.05).

HIV-1-specific T cell responses are known to decrease upon cART initiation, although not all responses and specificities may show similar decay kinetics [22], [23]. To determine whether maraviroc-intensified cART would lead to an equally rapid or even faster decay of global T cell responses to HIV-1, longitudinal changes in the breadth and magnitude of total HIV-1-specific T cell responses were compared between the maraviroc and control study arms at week 24 and week 60, i.e. 12 weeks after stopping maraviroc intensification. As previously reported, plasma viral load decreased under the limits of detection within the first 4-week cART in most patients [18]. CD4**^+^** T cell counts showed higher increases in the MVC subjects at week 12 and were significantly elevated in the MVC arm at week 60 when compared to the control subjects (p = 0.0378, Table 1 and ref [18]). At the same time, the decay in CD8^+^ T cells was significantly slower in MVC subjects than in the control subjects (Fig. 1A and [18]) To examine whether these effects on CD4^+^ and CD8^+^ T cell counts were associated with a modulation of T cell differentiation markers, the expression of CD45RA and CCR7 was assessed over time and compared between the two groups. The data show that the frequency of effector memory (EM, CD45RA^−^/CCR7^−^) CD8^+^ T cells was significantly decreased in both study arms at week 24 and week 60 compared to baseline, possibly reflecting the strong reduction in viral loads in both arms upon cART initiation (Fig. 1B). No significant changes for any other CD4^+^ or CD8^+^ T cell subset was observed, neither over time nor between study arms. These data indicate that maraviroc does not affect T cell differentiation during and after maraviroc intensification and that the different kinetics of CD4^+^ and CD8^+^ T cell counts between the arms are not reflected by gross alterations in differentiation markers.

### Maraviroc intensification is associated with maintenance of HIV-1-specific T cell responses

**Figure 2:**
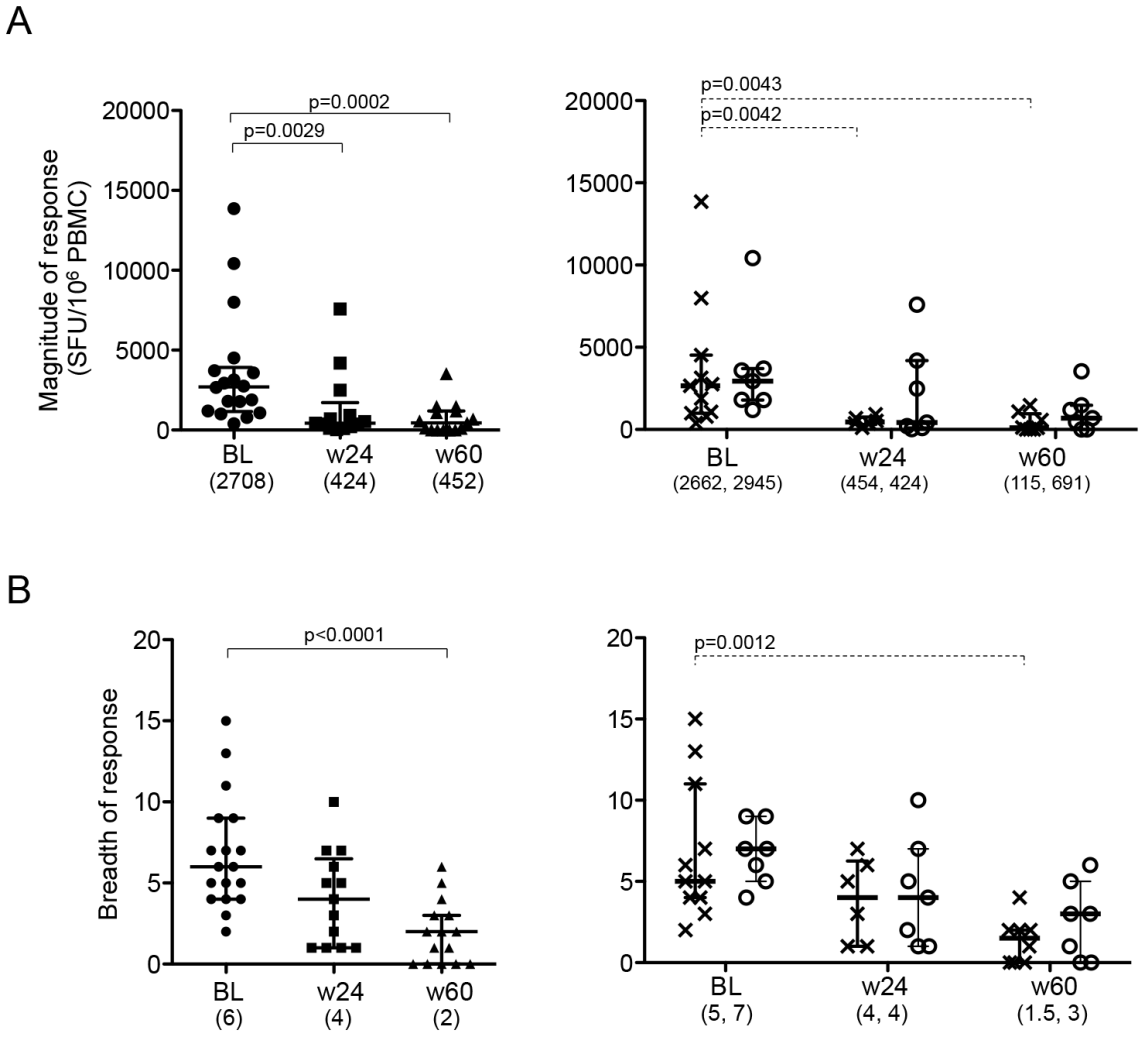
Longitudinal analyses of HIV-1-specific T cell responses in PBMC. The total magnitude (A) and breadth (B) of ELISpot responses at baseline (BL), week 24 (w24) and week 60 (w60) are shown for all subjects together (left panels) and for each study arm separately (right panels, crossed lines for control arm, circles for MVC arm). Horizontal lines represent median values of Spot-forming cells (SFC)/10^6^ PBMC and the IQR, respectively. Mann-Whitney test was used in all statistical analysis. Only p values with significance after Bonferroni correction was shown. The numbers in parenthesis below the x-axis represent the median value.

To assess whether the effect of maraviroc intensification on cell homeostasis affected the magnitude, breadth and specificity of the HIV-1-specific T cell response, we performed IFN-γ ELISPOT assay on PBMC from individuals in both arms of the study using a 18-mer overlapping peptide (OLP) set covering the full HIV-1 proteome [19]. At baseline, the median magnitude of HIV-1-specific T cell responses in all patients was 2,708 SFC/10^6^ PBMC (range 395–13,860), with a median breadth of 6 (range 2–15) responses per individual (Fig 2, left hand panels). The magnitude and the breadth in this cohort were considerably lower than that of chronically infected patients reported previously but in line with described breadth of responses in early, untreated HIV-1 infection [19], [24]. No significant difference was observed in magnitude and breadth of HIV-1-specific response between the arms at any time point (Fig. 2, right panels). When we assessed changes in the virus-specific response in each arm, the magnitude of the HIV-1-specific response in the control arm was significantly reduced by week 24 (median 454 SFC/10^6^ PBMC (range 27–7584), p = 0.0042) and even more so by week 60 (median 115 SFC/10^6^ PBMC (range 0–1,475), p = 0.0043, Fig 2A). In contrast, subjects in the MVC arm did not show a significant reduction until week 60 when their median magnitude was still more than 5-fold higher than responses in the control arm (median 691 SFC/10^6^ PBMC (range 0–3,535), Fig 2A). Similarly, the breadth of response was reduced over time as well, with significant reductions seen by week 60 in the control arm but not in the maraviroc intensified group (Fig 2B). There was no difference between the arms in regards to protein specificity of the HIV-1-specific CD8^+^ T cells that remained at 24 and 60 weeks after starting cART (data not shown).

**Figure 3:**
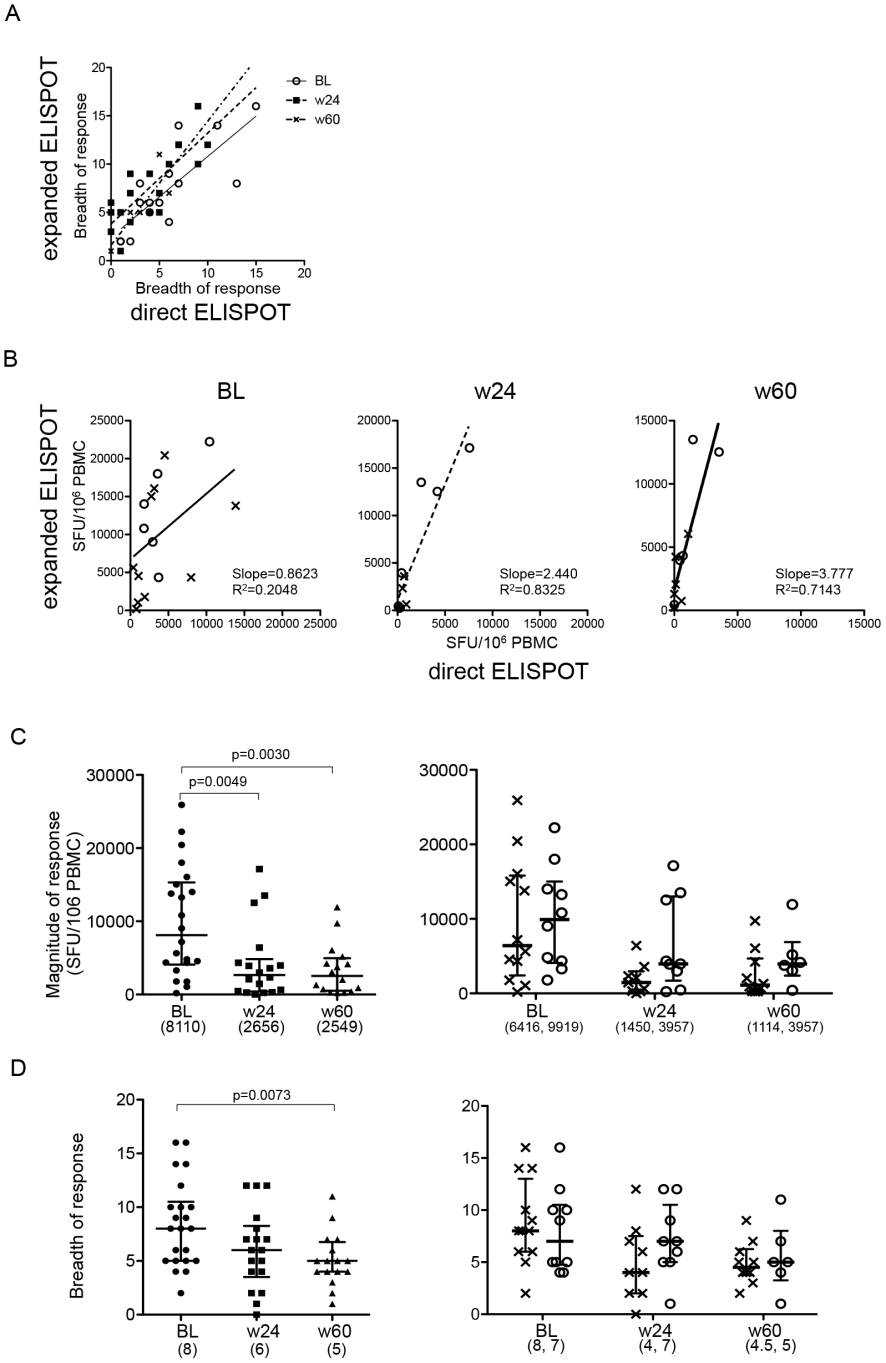
Longitudinal assessment of HIV-specific T cell responses with *in vitro* expanded T cells. A. Relationship of the breadth between responses detected by direct ELISpot and ELISpot using *in vitro* expanded cells. Responses on the x-axes represent the total HIV-1-specific responses in direct ELISpot, and the y-axes indicate total HIV-1-specific responses in expanded ELISpot for samples taken at baseline (circle), week 24 (square), and week 60 (triangle). B. Relationship of the magnitude between direct ELISpot and expanded ELISpot at each time point. cross: control arm, circle: MVC arm. The lines in A and B show linear regression lines. C, D. Changes in magnitude and breadth of total HIV-specific T cell responses in expanded ELISpot are shown as in Figure 2.

To extend the longitudinal analyses of responses between the intensified and non-intensified arms of the study to additional individuals for whom sample availability was limiting, we performed the same analysis using *in vitro* expanded cells. Aside from including additional individuals into the analyses, this also offered the opportunity to test for potential differences in the proliferative capacities of HIV specific T cells in the two arms. Thawed PBMC were expanded using an anti-CD3 mAb and kept in culture until sufficient cell numbers were reached. The culture time needed between the two study arms was comparable (both arms a median of 19 days), indicating intact proliferative capacities of T cells in maraviroc intensified cART treated individuals. For samples for which direct *ex-vivo* PBMC and *in vitro* expanded cells were tested, the ELISpot results were compared to validate the approach of using *in vitro*, unspecifically expanded cells. Overall, the breadth of responses in expanded cells correlated well with the direct *ex-vivo* results (Fig 3A, r = 0.78, p<0.0001). The magnitude of responses was generally increased in expanded cells, with later time points (week 24 and 60) showing stronger recovery of responses when compared to unexpanded cells (Fig. 3B). Of note, the correlation between results from direct *ex-vivo* analyses and *in-vitro* stimulated cells became stronger over time (r = 0.5235, 0.8455, 0.8720, and p = 0.0374, 0.0018, 0.0004 for comparisons at BL, w24, and w60 respectively). No differences were observed in proliferative capacity between the arms These data indicate that in both arms, HIV-1-specific T cell responses showed intact *in-vitro* proliferative capacities after prolonged cART and that in settings with limited sample availability, the *in-vitro* expansion approach produces reliable data [25].

HIV-1-specific T cell responses measured in expanded cells showed a significant decline in their magnitude during first 24 weeks in all subjects together (Fig. 3C, left panel). However, the reduction was generally less than three-fold (median 8,110 SFC/10^6^ PBMC in BL and 2,656 SFC/10^6^ PBMC in week 24) and thus not as dramatic as in unexpanded cells (median 6.3 fold, 2,708 SFC/10^6^ PBMC in BL, and 424 SFC/10^6^ PBMC in w24) (Fig. 2A and 3C, left panel). When the longitudinal changes in magnitude and breadth of responses were analyzed for each treatment arm separately, no significant reductions at week 24 and week 60 were noted (Fig 3C, D). However, when *in-vitro* stimulated responses were compared between the two arms, there was a trend that MVC-intensified subjects maintained stronger HIV-1-specific response at week 24 than control individuals (median 1,450 (IQR 277–2,965) in the control arm, 3,957 (1,714–13,018) in MVC, p = 0.0625, Fig. 3C, right panel). In addition, the median HIV-specific response was three-fold higher in MVC (median 3,957 (275–4,691)) compared to the control arm (1,114 (2,394–6,882)) until week 60. These data further support the notion that HIV-1-specific T cell responses are maintained for longer at higher levels in subjects with maraviroc intensification compared to individuals receiving non-intensified cART.

### Poly-functionality of HIV-1-specific CD8^+^ T cells is maintained under MVC intensified cART

**Figure 4:**
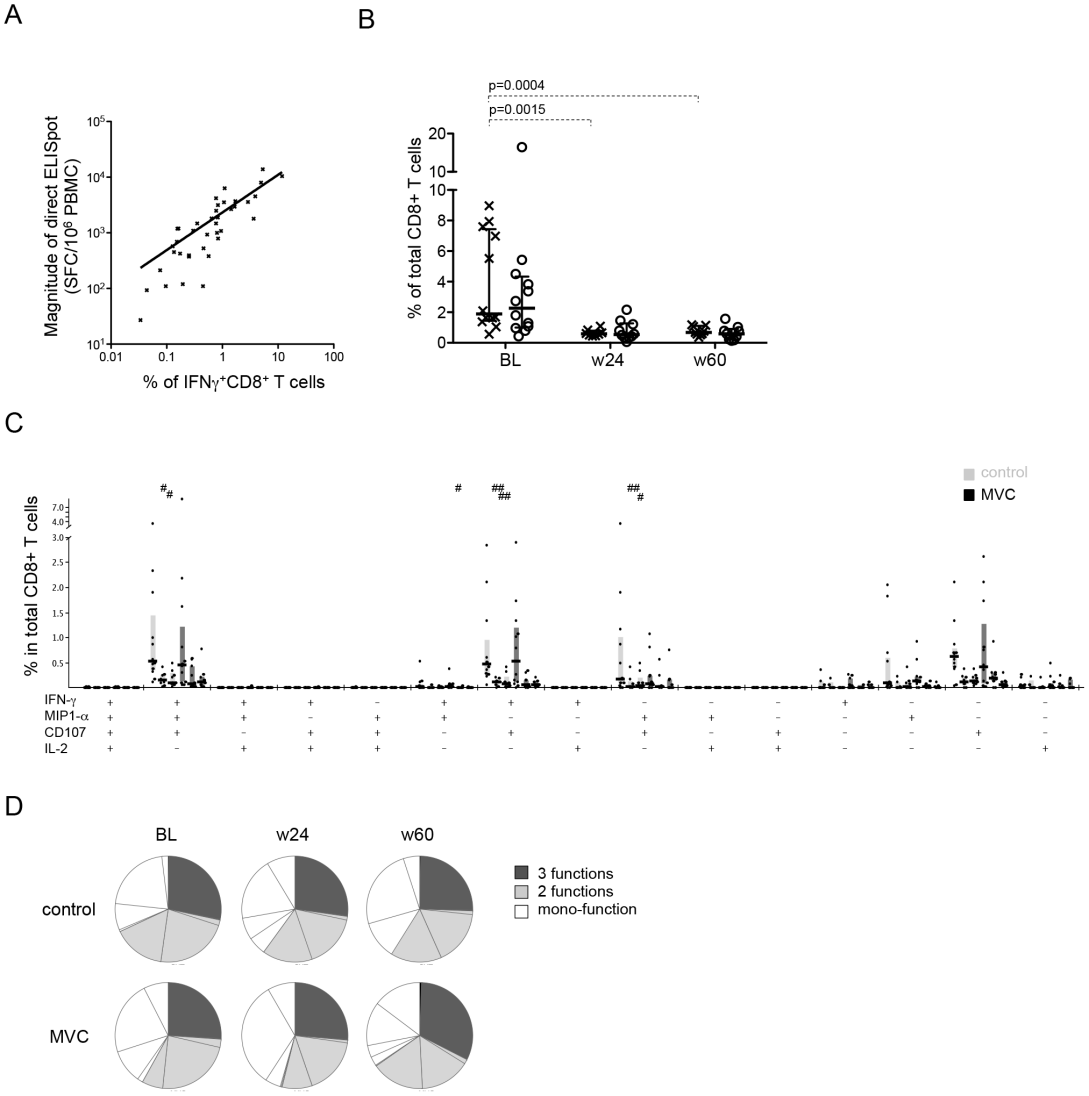
Longitudinal assessment in functional profile of HIV-specific CD8^+^ T cells during cART with MVC intensification. A. Correlation between the total HIV-specific responses determined by direct ex-vivo ELISpot analysis (as spot-forming cells (SFC)/10^6^ PBMC) and by ICS analysis (% of IFN-γ^+^ CD8^+^ T cells). Linear regression line, and correlation coefficient and p-values (Spearman's rank correlation test) are shown. B. The change in total HIV-specific CD8^+^ T cell frequency over time by ICS analysis. Horizontal lines indicate median values of all positive responses. P values were determined by Mann-Whitney tests and shown if the significance remains after Bonferroni correction. C, D. Effector function profiles of HIV-specific CD8^+^ T cells over time in controls and MVC treated subjects. (left, baseline; middle, week 24; right, week 60). The median and IQR are indicated by horizontal lines and boxes, respectively. Differences relative to baseline values in each arm were tested for statistic significance by Mann-Whitney tests, and shown as # for p<0.05, ## for p<0.01.

The ability of HIV-1-specific T-cells to respond to antigenic stimulus with multiple different effector functions has been associated with the relative control of HIV-1 infection [26], [27]. Since therapeutic strategies that aim at prolonged treatment interruptions or even viral eradication, will possibly depend on such polyfunctional T cell responses, we assessed the effector functions of HIV-1-specific CD8**^+^** T cells in cART treated subjects with and without maraviroc intensification. To this end, direct *ex-vivo* isolated PBMC were stimulated using peptide pools covering each of the viral proteins and analyzed for the expression of the degranulation marker (CD107a) or the production of intracellular cytokines, including IFN-γ, MIP-1β, and IL-2. The frequency of IFN-γ producing T cell responses correlated well with the data from the *ex-vivo* ELISpot analyses (Fig 4A, r = 0.8265, p<0.0001). The magnitude of the total HIV-1-specific CD8**^+^** T cell responses with at least one effector function by flow analysis varied widely in baseline samples (0.43% to 16.44% of total CD8**^+^** T cells across arms) and, as expected, was reduced at week 24 and week 60 (Fig. 4B). Although the magnitude of total HIV-specific CD8^+^ T cells between the arms was comparable at the different time points, a significant reduction in the strength of the ex-vivo response was seen in the control arm but not in MVC arm, as observed in direct *ex vivo* ELISpot analysis (Fig. 1A and 4B). Also, as the reduction in frequency of HIV-specific CD8^+^ T cell fractions with different cytokine secretion pattern was similar between the two arms, the data indicate that maraviroc intensification does not skew HIV-specific CD8^+^ T cell function (Fig. 4C). The same was observed when the relative contribution of T cell populations with different numbers of effecter functions to the total HIV-specific CD8 T cell responses was compared between arms and over time (Fig. 4D),in line with previous reports [26], [28].

## Discussion

Since its development as a HIV entry inhibitor, CCR5 has been used as a target in several clinical studies of HIV infection as well as in other applications, including auto-immune diseases, cancer and transplantation [15], [29]–[34]. Although some results remain still controversial [11]–[15], [17], [32] blocking the CCR5 co-receptor is thought to suppress adverse immune activation and inflammation by blocking the chemotactic activity via its inhibition of CCR5-mediated signals. Due to its potential immune-modulatory properties, maraviroc may thus also affect the HIV-specific immune response, not necessarily only in a beneficial manner. While a number of studies have described effects on total T cell counts, CD4**^+^** and CD8**^+^** T cell kinetics and outcome of vaccination to other pathogens [35]–[39], no study has, to our knowledge, investigated the effect of MVC on the total HIV-1-specific CD4**^+^** and CD8**^+^** T cell response. In the present study, we investigated the effect of maraviroc intensification on HIV-specific T cell responses in primary HIV-1 infected subjects treated with standard cART or maraviroc intensified regimen. Although there was no gross difference in specific T cell subsets, maraviroc intensification showed extended maintenance of stronger HIV-1-specific T cell responses when compared to non-intensified treatment in PBMCs.

Our data in recently infected and early treated individuals showed that maraviroc intensification accelerated recovery of CD4^+^ T cell counts and maintained higher CD4^+^ T cell count after its discontinuation (Table 1 and [18]). As CD4^+^ T cell help is critical for maintenance of memory CD8^+^ T cells [40], this early increase of CD4**^+^** T cells may also provide the basis for the extended maintenance of virus-specific T cell responses. Alternatively, the maintenance of higher HIV-1-specific T cell responses in maraviroc intensified subjects may be a reflection of a slower reduction in the total CD8^+^ T cells in the peripheral blood. This would be in line with clinical data showing that maraviroc intensification increase CD4^+^ T cells faster and reduce CD8^+^ T cell slower than non-intensified regimen (Fig. 1A and [18]). In addition, others have recently reported that maraviroc intensification increased CD8^+^ T cell counts in peripheral blood and decreased CD8^+^ T cells in rectal tissue in chronically HIV-infected subjects on stable cART [17], suggesting a possible *in vivo* redistribution of T cells by maraviroc. However, the relative changes of total CD8^+^ T cell counts between control arm and intensified group were less pronounced than the extensive changes in HIV-specific CD8**^+^** T cell frequencies, making it unlikely that a MVC-driven redistribution of virus-specific CD8**^+^** T cells would be the sole driving force behind the prolonged maintenance of these cells in the peripheral blood. Maintenance of virus-specific T cells has also be linked to the availability of cognate antigen[22]. As the reduction in virmeia in both arms was comparable, additional mechanisms may be at work in maraviroc-intensified individuals that lead to extended presence of cells. As shown in previous analyses, not all HIV-specific T cell response contract with the same kinetics and some even expand after cART initiation[23]. As there were no differences in the specificity of HIV-specific T cell response between the two arms in the present study, the mechanism for the maintenance of responses in the MVC intensified group remain unclear. One possibility is that the slower CD4 T cell decline in the intensified arm [18], together with a reported increased in T cell activation upon maraviroc intensification [17] maintains activated CD8 T cell for longer. Although a number of studies show conflicting data in terms of immune activation [11]–[15], it is important to note that the present data were generated in early treated subjects, which may yield different results than the analyses in maraviroc intensification during chronic HIV infection.

Blocking CCR5 signaling *in-vivo* might inhibit migration of memory T cells expressing CCR5 to the site of the cognate antigen, thus preventing these memory T cells to be stimulated properly to acquire effector functions and exert effective anti-viral immunity. In fact, maraviroc has been shown to inhibit chemotactic activity of lymphocytes and monocytes *in vitro* and to reduce the risk of Graft-versus-host disease (GVHD) in patients with hematologic cancers after allogeneic hematopoietic stem cell transplantation [16], [30], [41]. CCR5 polymorphisms and gene copy number of CCL3L, encoding one of CCR5's ligands, can affect delayed-type hypersensitivity (DTH) response [42], suggesting that CCR5 is critical for differentiation of CD8^+^ T cell, the acquisition of effector functions and the ability to traffic to the site of viral replication. However, we didn't observe any difference in T cell differentiation between the arms, and our data using directly isolated PBMC and *in vitro* expanded T cells indicate that the proliferative capacity of HIV-1-specific CD8**^+^** T cells in Maraviroc intensified subjects were not compromised. Furthermore, the effector function profiles were essentially identical between the two treatment arms, suggesting that maraviroc intensification does not negatively affect the quality of HIV-1-specific CD8**^+^** T cells. This is further supported by studies of the effects of maraviroc intensification on response to vaccination and immune profile in HIV-1 infected subjects [35]. Thus, studies of T cell effector function profiles in maraviroc intensified therapy, including their ability to mount HIV epitope-specific DTH reactions [43] may offer interesting insights into how maraviroc can modulate, and potentially improve, anti-viral immunity. In light of recent studies showing reduced viral reservoir sizes in MVC treated individuals [15] and data suggesting that a robust and functional HIV-1-specific CD8**^+^** T cell responses may be required for viral eradication strategies [44], a prolonged maintenance of functionally intact virus-specific T cells could provide the patient with a crucial advantage to further reduce the viral reservoir.

## References

[1] Samson M, Libert F, Doranz BJ, Rucker J, Liesnard C, et al (1996) Resistance to HIV-1 infection in caucasian individuals bearing mutant alleles of the CCR-5 chemokine receptor gene. Nature 382: 722–725. 8751444 10.1038/382722a0

[2] Liu R, Paxton WA, Choe S, Ceradini D, Martin SR, et al (1996) Homozygous defect in HIV-1 coreceptor accounts for resistance of some multiply-exposed individuals to HIV-1 infection. Cell 86: 367–377. 8756719 10.1016/s0092-8674(00)80110-5

[3] Paxton WA, Liu R, Kang S, Wu L, Gingeras TR, et al (1998) Reduced HIV-1 infectability of CD4+ lymphocytes from exposed-uninfected individuals: association with low expression of CCR5 and high production of beta-chemokines. Virology 244: 66–73. 9581779 10.1006/viro.1998.9082

[4] Glass WG, Lim JK, Cholera R, Pletnev AG, Gao JL, et al (2005) Chemokine receptor CCR5 promotes leukocyte trafficking to the brain and survival in West Nile virus infection. J Exp Med 202: 1087–1098. 16230476 10.1084/jem.20042530 PMC2213214

[5] Glass WG, McDermott DH, Lim JK, Lekhong S, Yu SF, et al (2006) CCR5 deficiency increases risk of symptomatic West Nile virus infection. J Exp Med 203: 35–40. 16418398 10.1084/jem.20051970 PMC2118086

[6] Camargo JF, Quinones MP, Mummidi S, Srinivas S, Gaitan AA, et al (2009) CCR5 expression levels influence NFAT translocation, IL-2 production, and subsequent signaling events during T lymphocyte activation. J Immunol 182: 171–182. 19109148 10.4049/jimmunol.182.1.171 PMC2937277

[7] Crawford A, Angelosanto JM, Nadwodny KL, Blackburn SD, Wherry EJ (2011) A role for the chemokine RANTES in regulating CD8 T cell responses during chronic viral infection. PLoS Pathog 7: e1002098. 21814510 10.1371/journal.ppat.1002098 PMC3141034

[8] Cooper DA, Heera J, Goodrich J, Tawadrous M, Saag M, et al (2010) Maraviroc versus efavirenz, both in combination with zidovudine-lamivudine, for the treatment of antiretroviral-naive subjects with CCR5-tropic HIV-1 infection. J Infect Dis 201: 803–813. 20151839 10.1086/650697

[9] Gulick RM, Lalezari J, Goodrich J, Clumeck N, DeJesus E, et al (2008) Maraviroc for previously treated patients with R5 HIV-1 infection. N Engl J Med 359: 1429–1441. 18832244 10.1056/NEJMoa0803152 PMC3078519

[10] Saag M, Goodrich J, Fatkenheuer G, Clotet B, Clumeck N, et al (2009) A double-blind, placebo-controlled trial of maraviroc in treatment-experienced patients infected with non-R5 HIV-1. J Infect Dis 199: 1638–1647. 19432546 10.1086/598965

[11] Cuzin L, Trabelsi S, Delobel P, Barbuat C, Reynes J, et al (2012) Maraviroc intensification of stable antiviral therapy in HIV-1-infected patients with poor immune restoration: MARIMUNO-ANRS 145 study. J Acquir Immune Defic Syndr 61: 557–564. 22986949 10.1097/QAI.0b013e318273015f

[12] Funderburg N, Kalinowska M, Eason J, Goodrich J, Heera J, et al (2010) Effects of maraviroc and efavirenz on markers of immune activation and inflammation and associations with CD4+ cell rises in HIV-infected patients. PLoS One 5: e13188. 20949133 10.1371/journal.pone.0013188 PMC2950842

[13] Wilkin TJ, Lalama CM, McKinnon J, Gandhi RT, Lin N, et al (2012) A Pilot Trial of Adding Maraviroc to Suppressive Antiretroviral Therapy for Suboptimal CD4+ T-Cell Recovery Despite Sustained Virologic Suppression: ACTG A5256. J Infect Dis 206: 534–542. 22740718 10.1093/infdis/jis376 PMC3491731

[14] Romero-Sanchez MC, Machmach K, Gonzalez-Serna A, Genebat M, Pulido I, et al (2012) Effect of Maraviroc on HIV Disease Progression-Related Biomarkers. Antimicrob Agents Chemother 56: 5858–5864. 22948867 10.1128/AAC.01406-12 PMC3486555

[15] Gutierrez C, Diaz L, Vallejo A, Hernandez-Novoa B, Abad M, et al (2011) Intensification of antiretroviral therapy with a CCR5 antagonist in patients with chronic HIV-1 infection: effect on T cells latently infected. PLoS One 6: e27864. 22174752 10.1371/journal.pone.0027864 PMC3234247

[16] Arberas H, Guardo AC, Bargallo ME, Maleno MJ, Calvo M, et al (2013) In vitro effects of the CCR5 inhibitor maraviroc on human T cell function. J Antimicrob Chemother 68: 577–586. 23152485 10.1093/jac/dks432

[17] Hunt PW, Shulman N, Hayes TL, Dahl V, Somsouk M, et al (2013) The immunologic effects of maraviroc intensification in treated HIV-infected individuals with incomplete CD4+ T cell recovery: a randomized trial. Blood 121: 4635–4646. 23589670 10.1182/blood-2012-06-436345 PMC3685899

[18] Puertas MC, Massanella M, Llibre JM, Ballestero M, Buzon MJ, et al.. (2013) Intensification of a raltegravir-based regimen with maraviroc in early HIV-1 infection. AIDS. In press. 10.1097/QAD.0000000000000066 24185044

[19] Frahm N, Korber BT, Adams CM, Szinger JJ, Draenert R, et al (2004) Consistent cytotoxic-T-lymphocyte targeting of immunodominant regions in human immunodeficiency virus across multiple ethnicities. J Virol 78: 2187–2200. 14963115 10.1128/JVI.78.5.2187-2200.2004 PMC369231

[20] Bihl FK, Loggi E, Chisholm JV 3rd, Hewitt HS, Henry LM, et al (2005) Simultaneous assessment of cytotoxic T lymphocyte responses against multiple viral infections by combined usage of optimal epitope matrices, anti- CD3 mAb T-cell expansion and “RecycleSpot”. J Transl Med 3: 20. 15888204 10.1186/1479-5876-3-20 PMC1164435

[21] Roederer M, Nozzi JL, Nason MC (2011) SPICE: exploration and analysis of post-cytometric complex multivariate datasets. Cytometry A 79: 167–174. 21265010 10.1002/cyto.a.21015 PMC3072288

[22] Spiegel HM, Chandwani R, Sheehy ME, Dobroszycki J, Fennelly G, et al (2000) The impact of early initiation of highly active antiretroviral therapy on the human immunodeficiency virus type 1-specific CD8 T cell response in children. J Infect Dis 182: 88–95. 10882585 10.1086/315639

[23] Gasser O, Brander C, Wolbers M, Brown NV, Rauch A, et al (2013) Expansion of interferon-gamma-secreting HIV-specific T cells during successful antiretroviral therapy. HIV Med 14: 241–246. 22934786 10.1111/j.1468-1293.2012.01040.x

[24] Altfeld M, Rosenberg ES, Shankarappa R, Mukherjee JS, Hecht FM, et al (2001) Cellular immune responses and viral diversity in individuals treated during acute and early HIV-1 infection. J Exp Med 193: 169–180. 11148221 10.1084/jem.193.2.169 PMC2193337

[25] Lecuroux C, Girault I, Boutboul F, Urrutia A, Goujard C, et al (2009) Antiretroviral therapy initiation during primary HIV infection enhances both CD127 expression and the proliferative capacity of HIV-specific CD8+ T cells. AIDS 23: 1649–1658. 19617814 10.1097/QAD.0b013e32832e6634

[26] Betts MR, Nason MC, West SM, De Rosa SC, Migueles SA, et al (2006) HIV nonprogressors preferentially maintain highly functional HIV-specific CD8+ T cells. Blood 107: 4781–4789. 16467198 10.1182/blood-2005-12-4818 PMC1895811

[27] Owen RE, Heitman JW, Hirschkorn DF, Lanteri MC, Biswas HH, et al (2010) HIV+ elite controllers have low HIV-specific T-cell activation yet maintain strong, polyfunctional T-cell responses. AIDS 24: 1095–1105. 20400885 10.1097/QAD.0b013e3283377a1e PMC2972651

[28] Lopez M, Soriano V, Rallon N, Cascajero A, Gonzalez-Lahoz J, et al (2008) Suppression of viral replication with highly active antiretroviral therapy has no impact on the functional profile of HIV-specific CD8(+) T cells. Eur J Immunol 38: 1548–1558. 18421792 10.1002/eji.200738054

[29] Fleishaker DL, Garcia Meijide JA, Petrov A, Kohen MD, Wang X, et al (2012) Maraviroc, a chemokine receptor-5 antagonist, fails to demonstrate efficacy in the treatment of patients with rheumatoid arthritis in a randomized, double-blind placebo-controlled trial. Arthritis Res Ther 14: R11. 22251436 10.1186/ar3685 PMC3392799

[30] Reshef R, Luger SM, Hexner EO, Loren AW, Frey NV, et al (2012) Blockade of lymphocyte chemotaxis in visceral graft-versus-host disease. N Engl J Med 367: 135–145. 22784116 10.1056/NEJMoa1201248 PMC3568501

[31] Nozza S, Pogliaghi M, Chiappetta S, Spagnuolo V, Fontana G, et al (2012) Levels of soluble endothelial protein C receptor are associated with CD4+ changes in Maraviroc-treated HIV-infected patients. PLoS One 7: e37032. 22715361 10.1371/journal.pone.0037032 PMC3371054

[32] Lisi L, Tramutola A, De Luca A, Navarra P, Dello Russo C (2012) Modulatory effects of the CCR5 antagonist maraviroc on microglial pro-inflammatory activation elicited by gp120. J Neurochem 120: 106–114. 22017448 10.1111/j.1471-4159.2011.07549.x

[33] Gramegna P, Latronico T, Brana MT, Di Bari G, Mengoni F, et al (2011) In vitro downregulation of matrix metalloproteinase-9 in rat glial cells by CCR5 antagonist maraviroc: therapeutic implication for HIV brain infection. PLoS One 6: e28499. 22174822 10.1371/journal.pone.0028499 PMC3234279

[34] Velasco-Velazquez M, Jiao X, De La Fuente M, Pestell TG, Ertel A, et al (2012) CCR5 antagonist blocks metastasis of basal breast cancer cells. Cancer Res 72: 3839–3850. 22637726 10.1158/0008-5472.CAN-11-3917

[35] Westrop SJ, Moyle G, Jackson A, Nelson M, Mandalia S, et al (2012) CCR5 Antagonism Impacts Vaccination Response and Immune Profile in HIV-1 Infection. Mol Med 18: 1240–1248. 22875102 10.2119/molmed.2012.00206 PMC3510292

[36] Cossarini F, Galli A, Galli L, Bigoloni A, Salpietro S, et al (2012) Immune recovery and T cell subset analysis during effective treatment with maraviroc. J Antimicrob Chemother 67: 2474–2478. 22678730 10.1093/jac/dks216

[37] Pulido I, Machmach K, Romero-Sanchez MC, Genebat M, Mendez-Lagares G, et al (2012) T-cell changes after a short-term exposure to maraviroc in HIV-infected patients are related to antiviral activity. J Infect 64: 417–423. 22227467 10.1016/j.jinf.2011.12.017

[38] Antoniou T, Smith G, Su D, Raboud JM, Lee D, et al (2012) Immunologic Effectiveness of Maraviroc- and Raltegravir-Containing Regimens (R+M+) versus Raltegravir-Based Regimens That Do Not Include Maraviroc (R+M-). J Int Assoc Physicians AIDS Care (Chic) 11: 192–197. 22247337 10.1177/1545109711424967

[39] Canestri A, Krivine A, Assoumou L, Le Corre M, Rozenberg F, et al (2010) Maraviroc does not affect humoral response to the pandemic influenza A-H1N1v 2009 adjuvanted vaccine in HIV-1-infected patients. AIDS 24: 2887–2889. 20962616 10.1097/QAD.0b013e3283402bc1

[40] Sun JC, Williams MA, Bevan MJ (2004) CD4+ T cells are required for the maintenance, not programming, of memory CD8+ T cells after acute infection. Nat Immunol 5: 927–933. 15300249 10.1038/ni1105 PMC2776074

[41] Rossi R, Lichtner M, De Rosa A, Sauzullo I, Mengoni F, et al (2011) In vitro effect of anti-human immunodeficiency virus CCR5 antagonist maraviroc on chemotactic activity of monocytes, macrophages and dendritic cells. Clin Exp Immunol 166: 184–190. 21985364 10.1111/j.1365-2249.2011.04409.x PMC3219893

[42] Dolan MJ, Kulkarni H, Camargo JF, He W, Smith A, et al (2007) CCL3L1 and CCR5 influence cell-mediated immunity and affect HIV-AIDS pathogenesis via viral entry-independent mechanisms. Nat Immunol 8: 1324–1336. 17952079 10.1038/ni1521

[43] Ruiz-Riol M, Mothe B, Gandhi RT, Bhardwaj N, Scadden DT, et al (2013) Influenza, but not HIV-specific CTL epitopes, elicits delayed-type hypersensitivity (DTH) reactions in HIV-infected patients. Eur J Immunol 43: 1545–1554. 23504637 10.1002/eji.201242732

[44] Shan L, Deng K, Shroff NS, Durand CM, Rabi SA, et al (2012) Stimulation of HIV-1-specific cytolytic T lymphocytes facilitates elimination of latent viral reservoir after virus reactivation. Immunity 36: 491–501. 22406268 10.1016/j.immuni.2012.01.014 PMC3501645

